# Successful treatment of plasma exchange for rapidly progressive interstitial lung disease with anti-MDA5 antibody-positive dermatomyositis: A case report

**DOI:** 10.1097/MD.0000000000010436

**Published:** 2018-04-13

**Authors:** Yushiro Endo, Tomohiro Koga, Takahisa Suzuki, Kazusato Hara, Midori Ishida, Yuya Fujita, Sosuke Tsuji, Ayuko Takatani, Toshimasa Shimizu, Remi Sumiyoshi, Takashi Igawa, Masataka Umeda, Shoichi Fukui, Ayako Nishino, Shin-ya Kawashiri, Naoki Iwamoto, Kunihiro Ichinose, Mami Tamai, Hideki Nakamura, Tomoki Origuchi, Masataka Kuwana, Atsushi Kawakami

**Affiliations:** a Department of Rheumatology, Unit of Advanced Preventive Medical Sciences, Graduate School of Bio-medical Sciences, Nagasaki University; b Department of Rheumatology, Japan Red Cross Nagasaki Genbaku Hospital, Nagasaki; c Department of Allergy and Rheumatology, Nippon Medical School Graduate School of Medicine, Tokyo, Japan.

**Keywords:** anti-MDA5 antibody, clinically amyopathic dermatomyositis, dermatomyositis, interstitial pneumonia, plasma exchange

## Abstract

**Rationale:**

As the initial treatment of rapidly progressive interstitial lung disease (RPILD) with antimelanoma differentiation-associated gene 5 antibody (anti-MDA5 Ab)-positive dermatomyositis (DM) patients, a combination of corticosteroids, cyclophosphamide, and calcineurin inhibitor is recommended. However, some of these patients have poor prognoses despite such intensive treatment. Other more effective treatments are desired. We report the case of an anti-MDA5 Ab-positive DM patient who had developed RPILD despite intensive treatments; she was treated successfully by a short-term plasma exchange (PE).

**Patient concerns:**

A 71-year-old Japanese woman was admitted to the rheumatology department of another hospital with progressive muscle weakness of the limbs and erythema on both upper eyelids and the fingers of both hands. She was suspected of having classical DM (CDM) based on the findings of typical skin and myositis. Although a chest computed tomography (CT) examination showed no findings of interstitial pneumonia at the first visit to the department, she newly presented interstitial pneumonia during her admission and her anti-MDA5 Ab titer was elevated.

**Diagnoses:**

She was diagnosed with interstitial lung disease (ILD) with anti-MDA5 Ab-positive DM.

**Interventions:**

She was treated with 1000 mg of methyl-prednisolone pulse, 500 mg of intravenous cyclophosphamide therapy (IVCY) followed by prednisolone 40 mg/day with tapering, and oral cyclosporine 200 mg/day. However, her interstitial pneumonia worsened with increasing breathing difficulty and an increasing serum ferritin level. She was transferred to our department, and we initiated PE as an additional treatment.

**Outcomes:**

After the PE treatment, all laboratory findings, for example, ferritin, KL-6, and the titer of anti-MDA5 Ab showed marked improvement, and the patient's skin symptoms and active interstitial pneumonia were relieved.

**Lessons:**

Our patient's case suggests that PE may be effective for RPILD in anti-MDA5 Ab-positive DM patients.

## 1 Introduction

Dermatomyositis (DM) characterized by amyopathy or hypomyopathy with typical skin symptoms is defined as clinically amyopathic dermatomyositis (CADM).^[1]^ Among CADM patients, antimelanoma differentiation-associated gene 5 antibody (anti-MDA5 Ab) was identified as a new autoantibody in 2005.^[2]^ The subsequent accumulation of anti-MDA5 Ab-positive patients revealed that anti-MDA5 Ab can also be detected among classical DM (CDM) patients satisfying the Bohan and Peter classification.^[3]^ These patients are known to frequently develop rapidly progressive interstitial lung disease (RPILD), with a poor prognosis.^[2,4]^

As the initial treatment of RPILD patients with anti-MDA5 Ab-positive DM, a combination of corticosteroids, cyclophosphamide, and calcineurin inhibitor is recommended.^[5]^ However, some of these patients have poor prognoses despite this intensive treatment.^[5]^ More effective treatments for RPILD patients with anti-MDA5 Ab-positive DM are strongly desired. In some cases, immunosuppressive treatments such as mycophenolate mofetil (MMF) and rituximab (RTX) may be effective for this disease,^[6,7]^ but the efficacy of these treatments has not yet been fully established.

Plasma exchange (PE) is an extracorporeal treatment that can remove certain pathologic substances such as circulating autoantibodies, cytokines, immune complexes, endotoxins, and other substances from the plasma.^[8]^ Since PE is highly effective for the removal of pathogenic autoantibodies with a half-life of approximately 21 days, it has demonstrated efficacy for refractory conditions in some autoimmune diseases since the 1980s.^[9]^ Although it can be speculated that the removal of anti-MDA Ab results in a favorable outcome of RPILD, little has been reported regarding the usefulness of PE for anti-MDA5 Ab-positive DM patients with RPILD.

We herein report the case of an anti-MDA5 Ab-positive DM patient who had developed RPILD despite the combination of corticosteroids, cyclophosphamide, and a calcineurin inhibitor; she was treated successfully with short-term PE.

## 2 Case report

In August 2017, a 71-year-old Japanese woman presented progressive muscle weakness of the limbs and erythema on both upper eyelids and the fingers of both hands. She was admitted to the rheumatology department of another hospital in late August 2017. On physical examination, tenderness and weakness in the proximal muscles of the limbs were observed, and she had typical skin symptoms such as erythemas on the nail circumference, upper eyelids, and both dorsal and palm sides around the proximal interphalangeal (PIP) and metacarpophalangeal (MCP) joints, suggesting heliotropic rash, Gottron sign, and inverse Gottron sign. The levels of creatinine kinase (CK) and ferritin were 720 IU/L (normal range 41–153 IU/L) and 2878 ng/mL (normal range 6.0–138 ng/mL) respectively in high titers. Magnetic resonance imaging (MRI) revealed markedly increased signal intensity of muscles in the lower limbs. Needle electromyography performed in the left vastus lateralis muscle, left tibialis anterior muscle, and left gastrocnemius muscle showed no obvious abnormalities. CDM was suspected based on the findings of skin and myositis.

A chest computed tomography (CT) examination showed no findings of interstitial pneumonia at the first visit to the rheumatology department. However, she newly had mild breathing difficulty at physical exertion when she was admitted to the hospital, and chest CT showed the development of interstitial pneumonia. Immediately after that, she showed a high titer of anti-MDA5 Ab (204 index).

She was diagnosed with interstitial lung disease (ILD) with anti-MDA5 Ab-positive DM. From mid-September, she was twice administered of 1000 mg of methyl-prednisolone pulse therapy and 500 mg of intravenous cyclophosphamide therapy (IVCY), followed by prednisolone 40 mg/day with tapering and oral cyclosporine 200 mg/day. Her symptoms including skin and interstitial pneumonia were improved. She was administered a 2nd 500 mg of IVCY again 3 weeks after the first administration, but immediately after that she suffered from cytomegalovirus (CMV) infection.

Although she was treated with ganciclovir (GCV), it was refractory and she was finally cured after the GCV was changed to foscarnet (FCN). However, from mid-November the interstitial pneumonia worsened with increasing breathing difficulty and increased levels of serum ferritin and Krebs von den lungen (KL)-6. The 3rd administration of methyl-prednisolone 1000-mg pulse therapy was not effective, and thus she was transferred to our department.

**Figure 1:**
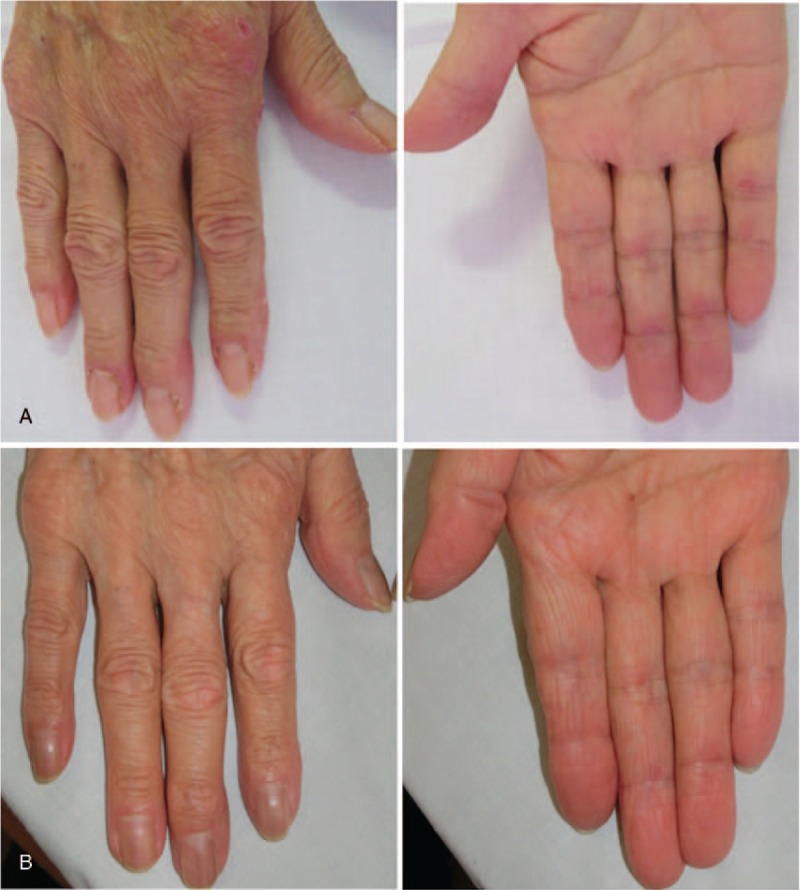
Erythemas and small ulcers on the nail circumference and erythemas on both dorsal and palm sides around the PIP and MCP joints (A). After the initiation of PE, the skin symptoms improved markedly (B). MCP = metacarpophalangeal, PE = plasma exchange, PIP = proximal interphalangeal.

On admission to our department, her body temperature was 36.2 °C, blood pressure was 125/79 mm Hg, heart rate was 105 beats/minute, and pulse oximetric saturation (SpO_2_) was 89% (room air). On physical examination, fine crackles were audible on the dorsal side of the bilateral lower lung regions, and she had erythemas on the nail circumference and both dorsal and palm sides around the PIP and MCP joints, suggesting Gottron sign and inverse Gottron sign, respectively. She also had small ulcers on the nail circumference (Fig. 1). Although she had no muscle pain, she had muscle weakness, and a manual muscle test showed 4 points out of 5 points in all proximal muscles of the upper and lower limbs.

Laboratory investigations showed the following results: white blood cell count (WBC) 8500 /μL (neutrophils 97.7%), hemoglobin (Hb) 11.6 g/dL, platelets (PLT) 23.2 × 10^4^ /μL, C-reactive protein (CRP) 1.08 mg/dL, lactate dehydrogenase (LDH) 394 IU/mL (normal range 124–222 IU/mL), ferritin 2878 ng/mL (normal range 6.0–138 ng/mL), and KL-6 2947 U/mL (normal range 105–401 U/mL). The CK level was 27 IU/L (within the normal range). No abnormalities were revealed by a urinalysis, and no liver or renal dysfunction was detected.

**Figure 2:**
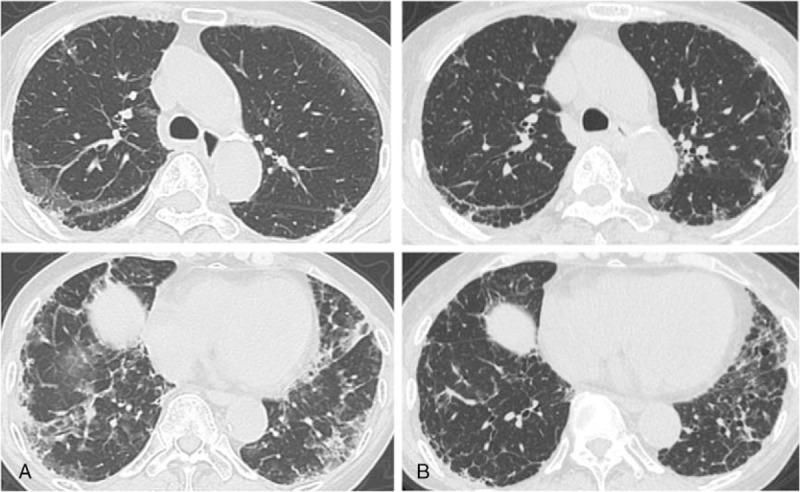
A chest CT on admission showed the expression of invasive shadows on the lung field under the pleura and on the dorsal side of the bilateral lower lobes (A), after the initiation of PE, the invasive shadows showed improvement (B). CT = computed tomography, PE = plasma exchange.

The following immunological and serological results were all negative (the exception is anti-MDA5 Ab): rheumatoid factor (RF), antinuclear antibody (ANA), proteinase-3 anti-neutrophil cytoplasmic autoantibodies (PR3-ANCAs), myeloperoxidase anti-neutrophil cytoplasmic autoantibodies (MPO-ANCAs), anti-ARS antibody, anti-transcription intermediary factor 1-gamma (TIF1-γ) antibody, and anti-Mi-2 antibody. The anti-MDA5 Ab titer index was 179 (normal range <32). The results of assays of β-D-glucan, T-SPOT.TB were all negative. A thoracico-abdominal CT examination showed the expression of invasive shadows on the lung field under the pleura and on the dorsal side of the bilateral lower lobes (Fig. 2A).

**Figure 3:**
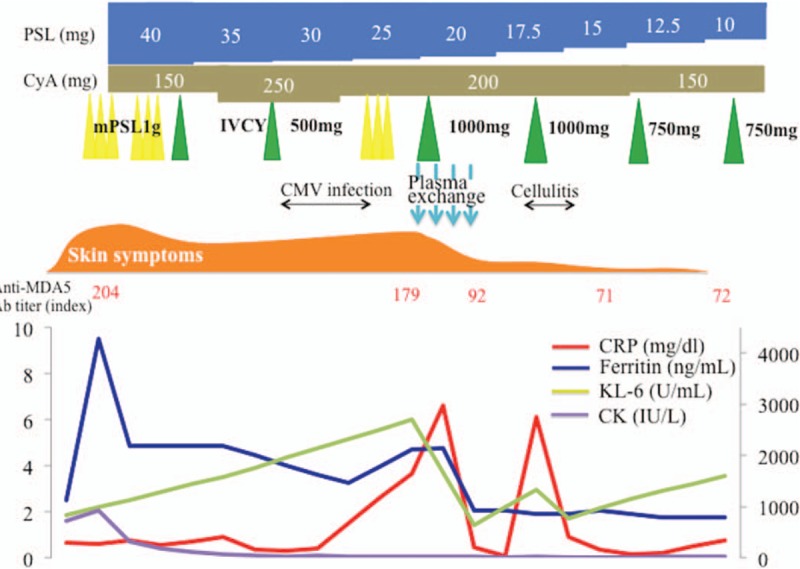
The clinical course of the patient, a 71-year-old Japanese woman. The CRP, the levels of ferritin, KL-6, and CK, and the treatment interventions during the hospital course are shown. CK = creatinine kinase, CMV = cytomegalovirus, CRP = C-reactive protein, CyA = cyclosporine, IVCY = intravenous cyclophosphamide therapy, KL = Krebs von den lungen, mPSL = methyl-prednisolone.

The patient's SpO_2_ at the rest was maintained at the 90% (room air) range, but at physical exertion it was easily decreased at the 80% (room air) range, and we started nasal oxygen therapy at the flow rate of 2.0 L/minute. Because her interstitial pneumonia progressed rapidly despite the combination of corticosteroids, cyclophosphamide, and calcineurin inhibitor, we initiated PE as an additional treatment at the following regimen: 3 times/week, fresh frozen plasma 30 units/time. We repeated 1000 mg of IVCY immediately after the first PE, which resulted in a marked improvement of all laboratory investigations including the serum levels of ferritin, KL-6, and the titer of anti-MDA5 Ab, the patient's skin symptoms, and the active interstitial pneumonia (Figs. 1B, 2B). The PE was completed in a total of 4 sessions, and after that the patient's breathing difficulty was gradually improved and she was able to withdraw from oxygen therapy. Early rehabilitation improved her muscle weakness, and we administered 3 more times of IVCY along with tapering of the dose of prednisolone, and the patient's remission has been maintained for over 2 months as of this writing (Fig. 3).

## 3 Discussion

Anti-MDA5 Ab-positive DM patients frequently develop RPILD, especially in East Asia.^[10]^ Reports of anti-MDA5 Ab-positive DM patients in Japan demonstrated the following prevalence: CADM, 85% (CDM, 15%); ILD, 92%; RPILD, 54%; and mortality, 46%,^[11]^ indicating that anti-MDA5 Ab-positive DM patients have poor prognoses. None of the previous reports showed a significant difference in mortality between anti-MDA5 Ab-positive CDM patients and anti-MDA5 Ab-positive CADM patients. It is still undetermined whether CADM is a distinct clinical entity or simply an early phase of CDM,^[12]^ but according to a report from the US, 33% of CADM patients at the first diagnosis developed CDM during mean 6.85 years of observation,^[13]^ thus suggesting the necessity of careful monitoring for the appearance of myositis in CADM patients.

The measurement of the anti-MDA5 Ab level is a novel tool for monitoring disease activity in RPILD with DM,^[14]^ and hyperferritinemia predicts poor prognosis, especially if the ferritin level is >1600 ng/mL.^[15]^ Therefore, these serum marker levels are very important when considering an earlier initiation of aggressive immunosuppressive therapies and enhancement of treatment. Our patient was unsuccessfully treated by a combination of corticosteroids, cyclophosphamide, and calcineurin inhibitor despite the early initiation of this intensive treatment. Because she developed RPILD with sequential increases of her levels of anti-MDA5 Ab and ferritin predicting a poor prognosis, it was necessary to administer another treatment to save her life.

Serum interleukin (IL)-6, IL-8, and IL-10 are associated with hyperferritinemia in RPILD with polymyositis (PM) or DM.^[16]^ Serum IL-6, IL-10, IL-18, and macrophage colony-stimulating factor (M-CSF) were significantly higher in anti-MDA5 Ab-positive DM patients than in anti-MDA5 Ab-negative DM patients before treatment, suggesting that the activation of monocytes and macrophages underlie the pathophysiology of anti-MDA5 Ab-positive DM patients.^[17]^ Among these cytokines, serum IL-18 is useful for the evaluation of response to treatment in ILD patients with anti-MDA5 Ab-positive DM.^[18]^ These reports suggest that a treatment that can reduce the anti-MDA5 Ab level and these various cytokines is expected to be effective for RPILD patients with anti-MDA5 Ab-positive DM. In our patient's case, these serum cytokines levels prior to the initial treatment were high compared to healthy individuals^[19]^ (Table 1).

**Table 1:**
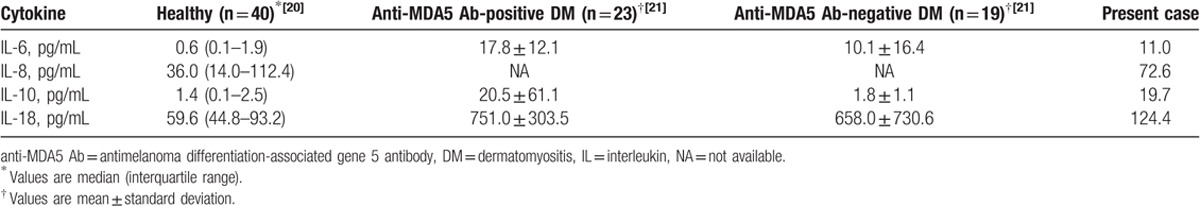
Comparison of cytokines in a healthy control, patients with anti-MDA5 Ab-positive DM, patients with anti-MDA5 Ab-negative DM, and the present patient.

Apheresis therapies are different from drug therapies in terms of the therapeutic mechanism for removing the etiology- and pathology-related substances from the plasma. Among apheresis therapies, PE can remove a wide range of substances (from large molecular weight to small molecular weight) by replacing the entirety of a patient's plasma with that of healthy persons. Short-term PE improved our patient's respiratory and skin symptoms with marked decreases of anti-MDA5 Ab and ferritin levels, and she has had a good prognosis. Although the direct pathogenicity of anti-MDA5 Ab has not been elucidated, the efficacy of PE in our patient's case may be due to the removal of anti-MDA5 Ab and various cytokines associated with hyperferritinemia and anti-MDA5 Ab-positive DM.

Since the earlier initiation of treatment is considered one of the prognostic factors in RPILD patients with anti-MDA5 Ab-positive DM,^[22]^ the time point of the appearance of therapeutic effects is important. Serum ferritin levels tend to decline approximately 2 weeks after the administration of IVCY, which is a key drug in the treatment of anti-MDA5 Ab-positive DM patients.^[17]^ Because an earlier initiation of the treatment affects the mortality rate, PE with immediate effects would be a better strategy for RPILD patients with anti-MDA5 Ab-positive DM until IVCY treatment is effective.

In our patient's case, we hesitated to strengthen immunosuppressive therapies, because we were concerned about the risk of relapse of the CMV infection which had been resistant to the treatment before she was transferred to our department. A CMV infection can cause severe damage to various organs (ie, intestine, lung, central nervous system, and eye), and the damage sometimes may be fatal – especially in immunocompromised patients.^[23]^ Considering that anti-MDA5 Ab-positive DM patients frequently develop RPILD with a poor prognosis and have a high risk of developing opportunistic infections caused by intensive immunosuppressive treatments, such infections will cause poorer prognoses for these patients. Therefore, even under such risks, PE is acceptable as an additional treatment and an alternative to immunosuppressive therapies.

DM is one of the autoimmune diseases that induce autoimmune-associated hemophagocytic syndrome (AAHS),^[24–26]^ and PE is one of the treatments for AAHS.^[24]^ However, the reported data related to the efficacy of apheresis therapy for ILD with polymyositis (PM) and DM are limited. Direct hemoperfusion using a polymyxin B-immobilized fiber column (PMX-DHP), which can reduce endotoxin levels and inflammatory chemical mediators such as cytokines,^[27]^ has demonstrated effectiveness for RPILD with CADM.^[21,28]^ In previous cases, PE was also effective for severe ILD with antiaminoacyl tRNA synthetase antibody-positive patients who were refractory to the standard treatments.^[20,29]^ Another case report showed the effectiveness of hemoperfusion with both PMX-DHP and PE for RPILD with anti-MDA5 Ab-positive CADM patients.^[30]^

In conclusion, we described a case of an anti-MDA5 Ab-positive DM patient who developed RPILD despite the combination of corticosteroids, cyclophosphamide, and a calcineurin inhibitor. She was treated successfully by short-term PE. Although PE has demonstrated efficacy for refractory conditions in autoimmune diseases, from the viewpoint of its immediate effectiveness and safety, PE should also be considered as a new treatment for RPILD patients with anti-MDA5 Ab-positive DM.

## Author contributions

**Writing – original draft:** Yushiro Endo.

**Supervision:** Tomohiro Koga, Takahisa Suzuki, Kazusato Hara, Midori Ishida, Yuya Fujita, Sosuke Tsuji, Ayuko Takatani, Toshimasa Shimizu, Remi Sumiyoshi, Takashi Igawa, Masataka Umeda, Shoichi Fukui, Ayako Nishino, Shin-ya Kawashiri, Naoki Iwamoto, Kunihiro Ichinose, Mami Tamai, Hideki Nakamura, Tomoki Origuchi, Masataka Kuwana, Atsushi Kawakami.
